# Phase I study of the anti-FcRH5 antibody-drug conjugate DFRF4539A in relapsed or refractory multiple myeloma

**DOI:** 10.1038/s41408-019-0178-8

**Published:** 2019-02-04

**Authors:** A. Keith Stewart, Amrita Y. Krishnan, Seema Singhal, Ralph V. Boccia, Manish R. Patel, Ruben Niesvizky, Asher A. Chanan-Khan, Sikander Ailawadhi, Jochen Brumm, Kirsten E. Mundt, Kyu Hong, Jacqueline McBride, Quyen Shon-Nguyen, Yuanyuan Xiao, Vanitha Ramakrishnan, Andrew G. Polson, Divya Samineni, Douglas Leipold, Eric W. Humke, James Scott McClellan, Jesus G. Berdeja

**Affiliations:** 1 Division of Hematology-Oncology, Mayo Clinic, Phoenix, AZ USA; 2 Judy and Bernard Briskin Center for Multiple Myeloma Research, City of Hope Medical Center, Duarte, CA USA; 3 Lurie Comprehensive Cancer Center, Northwestern University, Chicago, IL USA; 4 Center for Cancer and Blood Disorders, Bethesda, MD USA; 5 Florida Cancer Specialists, Sarasota, FL USA; 6 Sarah Cannon Research Institute, Nashville, TN USA; 7 Multiple Myeloma Center, New York Presbyterian Hospital-Cornell Medical Center, New York, NY USA; 8 Division of Hematology & Oncology, Mayo Clinic, Jacksonville, FL USA; 9 Genentech, Inc., South San Francisco, CA USA

## Abstract

FcRH5 is a cell surface marker enriched on malignant plasma cells when compared to other hematologic malignancies and normal tissues. DFRF4539A is an anti-FcRH5 antibody-drug conjugated to monomethyl auristatin E (MMAE), a potent anti-mitotic agent. This phase I study assessed safety, tolerability, maximum tolerated dose (MTD), anti-tumor activity, and pharmacokinetics of DFRF4539A in patients with relapsed/refractory multiple myeloma. DFRF4539A was administered at 0.3–2.4 mg/kg every 3 weeks or 0.8–1.1 mg/kg weekly as a single-agent by intravenous infusion to 39 patients. Exposure of total antibody and antibody-conjugate-MMAE analytes was linear across the doses tested. There were 37 (95%) adverse events (AEs), 8 (21%) serious AEs, and 15 (39%) AEs ≥ grade 3. Anemia (*n* = 10, 26%) was the most common AE considered related to DFRF4539A. Two cases of grade 3 acute renal failure were attributed to DFRF4539A. There were no deaths; the MTD was not reached. DFRF4539A demonstrated limited activity in patients at the doses tested with 2 (5%) partial response, 1 (3%) minimal response, 18 (46%) stable disease, and 16 (41%) progressive disease. FcRH5 was confirmed to be expressed and occupied by antibody post-treatment and thus remains a valid myeloma target. Nevertheless, this MMAE-based antibody-drug-conjugate targeting FcRH5 was unsuccessful for myeloma.

## Introduction

Several classes of drugs including immunomodulatory agents^1^, monoclonal antibodies^2^, and proteasome inhibitors^3^ have shifted the paradigm of treatment choices and outcomes for multiple myeloma patients. Newer agents such as daratumumab, bortezomib, carfilzomib, lenalidomide, and pomalidomide have contributed towards better survival outcomes^4^. However, even with improved treatments, the majority of multiple myeloma patients continue to experience relapse. There is thus a continued need for treatments that may significantly extend disease-free and overall survival, with desirable safety profiles. A recently identified gene family that encodes cell surface receptors on B cells has provided a potential target for new drug development. Fc receptor-homolog 5 (FcRH5; also known as FcRL5, IFGP5, BXMAS1, CD307, and IRTA2)^5,6^ belongs to a family of six genes of the immunoglobulin superfamily (IgSF)^7^. FcRH5 is a cell surface antigen of unknown function whose expression is restricted to mature B cells, and expression is maintained on plasma cells (PCs). Notably, as compared to normal human PCs, FcRH5 is expressed at higher levels in multiple myeloma cells^7–9^.

DFRF4539A is an antibody-drug conjugate (ADC) that contains a humanized immunoglobulin-G1 (IgG1) anti-human FcRH5 monoclonal antibody (MFRF3266A) and a potent anti-mitotic agent, monomethyl auristatin E (MMAE), linked through a protease-labile linker, maleimidocaproyl-valine-citrulline-p-aminobenzyloxycarbonyl (MC-VC-PABC)^8^. In preclinical models, antibodies bound to FcRH5 were internalized, suggesting that FcRH5 may be suited for targeted delivery of cytotoxic agents^8,10^. MMAE has a mode of action similar to vincristine, and ADCs containing MMAE have induced durable responses in hematologic malignancies including Hodgkin lymphoma^11,12^. Following internalization, the conjugate is cleaved by lysosomal enzymes to release MMAE, which binds to tubulin and disrupts the microtubule network, thereby resulting in inhibition of cell division and cell growth^13–15^. DFRF4539A has demonstrated efficacy in nonclinical xenograft models of human FcRH5-positive multiple myeloma^8^, and based on this data and an acceptable nonclinical safety profile, we tested this agent in patients with relapsed/refractory multiple myeloma in a phase I clinical trial.

## Methods

### Study design

This phase I open-label study (ClinicalTrials.gov number NCT01432353) was designed to investigate the safety, tolerability, pharmacokinetics (PK), and biologic activity of DFRF4539A (supplied by Genentech, Inc.) in patients with relapsed/refractory multiple myeloma, and to determine a recommended phase II dose (RP2D). DFRF4539A was administered at doses of 0.3–2.4 mg/kg every 3 weeks or 0.8–1.1 mg/kg weekly as a single agent by intravenous (IV) infusion. The study consisted of a dose-escalation stage using a 3 + 3 design to determine the maximum tolerated dose (MTD) of an every 3 week (Q3W) dosing schedule, followed by a cohort expansion at the Q3W RP2D to further characterize the safety and activity of DFRF4539A. Additionally, to test whether more frequent dosing would be better tolerated, a weekly (Q1W) dosing schedule was implemented to determine the MTD of weekly dosing.

### Patients

Eligible patients age ≥ 18 years had relapsed or refractory multiple myeloma for which no effective standard therapy exists. Patients were required to have been previously treated with a proteasome inhibitor or immunomodulatory drug. Eastern Cooperative Oncology Group (ECOG) performance status of 0–2, measurable disease as per the International Myeloma Working Group definitions^16^ by the presence of plasma or urine monoclonal protein, and serum immunoglobulin free light chain (FLC) and abnormal serum immunoglobulin kappa (FLC to lambda FLC ratio) were also required. Patients receiving monoclonal antibody within 4 weeks of planned cycle 1, day 1 (C1D1), radio/chemotherapy with 2 weeks of C1D1, autologous stem cell transplant within 100 days prior to C1D1, prior allogenic stem cell transplant, or diagnosed with confounding malignancy were excluded.

The protocol was approved by Institutional Review Boards prior to patient recruitment and was conducted in accordance with International Conference on Harmonization E6 Guidelines for Good Clinical Practice. Written informed consent was obtained for all patients prior to performing study-related procedures in accordance with federal and institutional guidelines.

### Safety assessment

The primary objective of the study was to assess safety and tolerability of DFRF4539A. Safety was evaluated according to NCI CTCAE v4.0. Dose limiting toxicities (DLT) were defined as grade 3–4 non-hematologic toxicity, except for reversible grade 3 allergic and non-allergic infusion toxicities, grade 3 or 4 hypercalcemia if due to underlying disease, grade 3 or 4 hyperuricemia, hyperphosphatemia, or hypocalcemia or grade 3 hyperkalemia, if transient (i.e., lasting <48 h) and without manifestations of clinical tumor lysis syndrome (i.e., creatinine ≥ 1.5 × the upper limit of normal [ULN], cardiac arrhythmias, sudden death, or seizures). The following hematologic toxicities also qualified as DLTs unless they were related to underlying disease: grade 4 anemia, grade 3 or 4 thrombocytopenia, and grade 3 or 4 neutropenia that did not recover to grade ≤2 within 72 h without growth factor support or was accompanied by temperature elevation (oral or tympanic temperature of ≤38 °C). The MTD was defined as the highest dose at which ≤17% of patients at an assigned dose experienced a protocol-defined DLT.

### Pharmacokinetics and immunogenicity assessments

Serum concentrations of total antibody (a measure of three key antibody analytes: conjugated, partially de-conjugated, and fully de-conjugated antibody) was determined using a validated enzyme-linked immunosorbent assay (ELISA)^17,18^. DFRF4539A conjugate (as antibody-conjugated MMAE [ac-MMAE]) concentrations were measured in plasma samples by protein A affinity capture followed by enzyme-mediated release of MMAE and analysis using liquid chromatography-tandem mass spectrometry (LC-MS/MS^17,18^.

PK parameters for the total antibody, ac-MMAE, and unconjugated MMAE following the first dose of DFRF4539A in cycle 1 (Q3W and weekly schedules) were estimated using non-compartmental analysis using WinNonlin 5.2.1 software (Pharsight, Sunnyvale, CA). Baseline and pre-dose post-baseline serum anti-drug antibody (ADA) samples were collected from all treated patients, and analyzed using a validated bridging antibody ELISA to screen, confirm and characterize ADA responses^19,20^. Confirmed positive ADA responses were further characterized by competitive binding to determine if the response was primarily directed against the antibody or the linker-drug portion of the ADC and the relative ADA levels determined by titration.

### Preliminary assessment of anti-tumor activity

Objective response, defined as a stringent complete, complete, very good partial, partial response, or minimal response, confirmed ≥4 weeks after initial documentation was determined using International Myeloma Working Group (IMWG) Uniform Response Criteria and/or European Bone Marrow Transplant (EBMT) Criteria^21,22^. Duration of objective response was defined as the time from first occurrence of a documented, objective response until the time of relapse or death from any cause. Progression-free survival (PFS) was defined as the time elapsed between treatment initiation and tumor progression or death from any cause.

### Biomarker assessment

FcRH5 (CD307) expression and occupancy were assessed using a validated flow cytometry method (Labcorp, Burlington, North Carolina, USA) as described previously^8,10^. Cells were stained with appropriate antibody combinations and data was acquired on FACSCanto II (BD BioSciences, San Jose, CA). Briefly, 100 µL of sodium heparin anticoagulated whole blood or bone marrow from each patient per available time point, shipped at 3–8 °C for receipt within 54 h of collection, was pipetted into test tubes. Five µL of human serum (Sigma, St. Louis, MO) and 5 µL of mouse serum (Rockland Immunochemicals, Limerick, PA) were added to the cells and incubated for 10 min at room temperature. The cells were stained with the appropriate fluorescent-conjugated antibody combinations (Ms IgG_1_, CD27, humanized anti-gD, Her2, FcRH5 [10A8], FcRH5 [7D11], CD19, CD138, CD3, CD38, CD45, and CD56) (BD Biosciences, San Jose, CA; Southern Biotech, Birmingham, AL; Invitrogen/Thermo Fisher Scientific, Waltham, MA) and incubated on ice for 30 min in the dark. All tubes were lysed with 4 mL of a cold (2–8 °C) ammonium chloride lysing solution (BD Biosciences) and washed with 2 mL phosphate buffered saline (PBS) (BD Biosciences) with 1% bovine serum albumin (BSA) (Hyclone/GE Healthcare Life Sciences, Logan, UT). All cells were then stained with the appropriate amount of Streptavidin-Qdot605 (Invitrogen) on ice for 20 min in the dark. The cells were washed again with PBS with 1% BSA, resuspended in 500 µL of 1% paraformaldehyde solution (BD Biosciences) and stored at 2–8 °C until they were acquired on the FACSCantoII. Fluorescent quantitation beads (BD Biosciences) were acquired daily for quality control of the instruments. PCs were defined by strong CD138 and CD38 expression, CD45 Lo, and with light scatter characteristics of large mononuclear cells. Gated PCs were used to calculate FcRH5 expression and drug occupancy; anti-Her2 was used as a negative control. Fluorescence intensity was quantified using standard units—molecule of equivalent soluble fluorophores (MESF).

### Statistical analysis

Design considerations were not made with regard to explicit power and type I error, but to obtain preliminary safety, PK, and PD information. All patients who received any amount of DFRF4539A were included in the safety-evaluable population. For activity analyses, all patients who completed at least one on-treatment disease assessment were included.

## Results

### Baseline patient demographics and treatment

Thirty-nine patients were enrolled at 8 sites between 17 September 2011, and 7 April 2014. The cutoff date for analysis was 2 September 2014, resulting in a median follow-up time of 43 days (range 16–241 days) for 39 patients. Patients demonstrated uniform baseline and disease characteristics (Table 1). All patients had undergone prior systemic therapy (median six prior therapies, range 2–13), and all had previously received proteasome and corticosteroid therapy. The majority of patients had previously undergone immunomodulatory therapy (97%) as well. The patient population thus represented a multiple treatment relapse population with significant unmet need.

**Table 1:** Baseline and disease characteristics by dose group

	Q3W dosing					Q1W dosing		All patients (*N* = 39)
	0.3 mg/kg (*n* = 3)	0.6 mg/kg (*n* = 3)	1.2 mg/kg (*n* = 3)	1.8 mg/kg (*n* = 7)	2.4 mg/kg (*n* = 17)	0.8 mg/kg (*n* = 3)	1.1 mg/kg (*n* = 3)	
Age (yr), median (range)	62 (52−73)	66 (51−71)	58 (56−62)	65 (51−73)	63 (48−75)	69 (42−80)	63 (59−78)	63 (42−80)
Sex, male	1 (33%)	2 (67%)	3 (100%)	4 (57%)	9 (53%)	1 (33%)	1 (33%)	21 (54%)
ECOG performance status, median (range)	1 (1−1)	1 (1−1)	1 (0−1)	1 (0−2)	1 (0−2)	1 (0−1)	1 (0−1)	1 (0−2)
Duration of malignancy (months), median (range)	91 (46−101)	88 (47−116)	93 (48−100)	79 (11−106)	92 (8−141)	54 (37−125)	26 (21−171)	88 (8−171)
Prior cancer treatment, Systemic therapy, median (range)								
Systemic therapy	8 (7−9)	6 (5−6)	9 (5−10)	4 (2−10)	5 (2−13)	7 (3−8)	4 (3−7)	6 (2−13)

*ECOG* Eastern Cooperative Oncology Group

The study initially enrolled patients on the Q3W dose-escalation schedule (Supplementary Figure S1). During escalation, the first DLT occurred among the first three patients enrolled in the 1.8 mg/kg cohort. Consequently, an additional four patients were enrolled at 1.8 mg/kg. No further DLTs were observed at this dose level; therefore, the escalation continued to 2.4 mg/kg. At this dose and schedule, a DLT was observed in one of the first three patients, and an additional three patients were enrolled. No further DLTs were observed at the 2.4 mg/kg dose level. However, based on the totality of the safety data, this dose was determined to be the RP2D and cohort expansion occurred for this dose and schedule, enrolling 11 additional patients. Data for this dose and schedule were combined from both the dose-escalation and expansion patients (*n* = 17).

Once the Q3W RP2D was declared (2.4 mg/kg), a Q1W dose-escalation cohort was opened, starting at a dose of 0.8 mg/kg and escalating to a dose of 1.1 mg/kg Q1W with three patients per cohort. No DLTs were observed in either cohort. However, following a review of the safety, tolerability, PK, and activity, no further enrollment on the Q1W schedule was pursued.

The MTD was not reached in this study. Overall, the median duration of exposure to DFRF4539A among patients was 22 days (range 1−219) (Supplementary Figure S2). The number of treatment cycles received ranged from 1 to 11 (median 2). All 39 enrolled patients discontinued the study. The majority of patients were withdrawn due to disease progression (29 patients [74%]). Seven patients (18%) were withdrawn because of AEs, and three patients (8%) were withdrawn at the investigator’s discretion.

### Safety

Adverse events (AEs) related to DFRF4539A are shown in Supplementary Table S1, the most common of which were anemia (26%) and fatigue (21%). Twelve patients (31%) experienced grade 3 AEs, and three patients (8%) experienced grade 4 AEs (Table 2). Nine patients (23%) experienced at least one AE related to DFRF4539A with grade ≥ 3 intensity. The most common grade ≥ 3 AE deemed related to study drug was neutropenia (four patients [10%]); two patients (one each in the 0.3 mg/kg Q3W cohort and the 2.4 mg/kg Q3W cohort) experienced grade 3 neutropenia, and two patients (both in the 2.4 mg/kg Q3W cohort) reported grade 4 neutropenia.

**Table 2:** All adverse events grade ≥3 occurring in ≥2 patients

	Q3W dosing					Q1W dosing		All patients (*N* = 39)
	0.3 mg/kg (*n* = 3)	0.6 mg/kg (*n* = 3)	1.2 mg/kg (*n* = 3)	1.8 mg/kg (*n* = 7)	2.4 mg/kg (*n* = 17)	0.8 mg/kg (*n* = 3)	1.1 mg/kg (*n* = 3)	
Any AE	1 (33.3%)	2 (66.7%)	0	1 (14.3%)	8 (47.1%)	1 (33.3%)	2 (66.7%)	15 (38.5%)
Infections and infestations	1 (33.3%)	1 (33.3%)	0	0	0	1 (33.3%)	1 (33.3%)	4 (10.3%)
Neutropenia	1 (33.3%)	0	0	0	3 (17.6%)	0	0	4 (10.3%)
Nervous system disorders	0	1 (33.3%)	0	0	2 (11.8%)	0	0	3 (7.7%)
Alanine amino-transferase increased	0	0	0	1 (14.3%)	1 (5.9%)	0	0	2 (5.1%)
Anemia	0	0	0	0	1 (5.9%)	0	1 (33.3%)	2 (5.1%)
Aspartate amino-transferase increased	0	0	0	1 (14.3%)	1 (5.9%)	0	0	2 (5.1%)
Hyperglycemia	0	0	0	1 (14.3%)	1 (5.9%)	0	0	2 (5.1%)
Hyponatremia	0	0	0	0	2 (11.8%)	0	0	2 (5.1%)
Musculo-skeletal and connective tissue disorder	0	1 (33.3%)	0	0	1 (5.9%)	0	0	2 (5.1%)
Renal failure acute	0	0	0	0	2 (11.8%)	0	0	2 (5.1%)
Thrombo-cytopenia	0	0	0	0	2 (11.8%)	0	0	2 (5.1%)

In total, eight patients (21%) experienced serious AEs (SAEs) regardless of relationship to study drug (Supplementary Table 2). One patient experienced a grade 2 hypercalcemia, seven patients experienced grade 3 SAEs; grade 3 SAEs included acute renal failure, back pain, pathological fracture, and pneumonia. Two patients (5%) receiving DFRF4539A on the 2.4 mg/kg Q3W dosing schedule experienced acute renal failure (ARF). These two SAEs were deemed related to the study drug. The remaining grade 3 SAEs occurred in one patient (3%) each. There were no deaths on the study.

One DLT of grade 3 transaminitis was observed at 1.8 mg/kg Q3W. At 2.4 mg/kg Q3W, one DLT was observed that consisted of grade 3 hyponatremia accompanied by altered mental status (metabolic encephalopathy) requiring ICU admission, ARF, thrombocytopenia, and neutropenia.

Peripheral neuropathy is a known side effect of MMAE-linked ADCs^12^, and peripheral neuropathy was observed in eight patients (21%) treated with DFRF4539A. The median time to onset of peripheral neuropathy at the 2.4 mg/kg Q3W dosing schedule was 105 days (95% CI, 71-NE) (Supplementary Figure S3).

Per protocol, pre-infusion prophylactic medications including corticosteroids were not allowed prior to the first DFRF4539A infusion. No episodes of hypersensitivity to DFRF4539A were reported, and consequently, corticosteroids were not administered in subsequent doses.

### Pharmacokinetics

DFRF4539A total antibody and ac-MMAE demonstrated linear PK across the tested doses of 0.8–2.4 mg/kg doses at both the Q3W and the Q1W schedules. The average C_max_ and AUC_inf_ values of total antibody were higher, t_1/2_ values were longer, and CL values were lower than those of ac-MMAE compared within the same dose group in both the Q3W and Q1W schedules. The V_ss_ values for the ac-MMAE and total antibody analytes approximated the physiological plasma volume and were generally independent of dose, suggesting that the ac-MMAE distribution was dominated by its antibody component. The systemic unconjugated MMAE exposure was consistently low (>100-fold less than exposure to ac-MMAE), suggesting that majority of MMAE remained conjugated to the ADC in circulation. The *T*_max_ of unconjugated MMAE was delayed as compared to ac-MMAE; t_1/2_ of unconjugated MMAE was relatively long for a small molecule drug and was approximate towards that of ac-MMAE. These results suggested that the slow and sustained release of unconjugated MMAE and its elimination rate was limited by its formation rate. Minimal accumulation was observed for all the three analytes upon repeated dosing for the Q3W and Q1W schedules, suggesting steady state was reached within cycle 1.

The PK parameters for all the three analytes at the RP2D dose level of 2.4 mg/kg at the Q3W schedule are summarized in Table 3. The cumulative exposure of acMMAE and total antibody in cycle 1 after the weekly dose of 0.8 mg/kg was roughly similar to the exposure observed at Q3W at 2.4 mg/kg in cycle 1.

**Table 3:** Mean (% CV) for DFRF4539A pharmacokinetic parameters—including total antibody, antibody-conjugated mono methyl auristatin E (ac-MMAE), and unconjugated MMAE analytes—in cycle 1 at 2.4 mg/kg

Dose (mg/kg)	*C*_max_ Mean (%CV)			AUC_inf_ Mean (%CV)			CL Mean (%CV)		
	Total Ab (μg/mL)	ac-MMAE (ng/mL)	Unconjugated MMAE (ng/mL)	Total Ab (μg/mL)	ac-MMAE (ng/mL)	Unconjugated MMAE (ng/mL)	Total Ab (μg/mL)	ac-MMAE (ng/mL)	Unconjugated MMAE (ng/mL)
2.4 Q3W (*n* = 16)	55.1 (32%)	876 (49%)	3.14 (45%)	291 (35%)	2660 (33%)	35.2 (54%)	9.6 (44%)	17.6 (37%)	NA

*AUC*_*inf*_ area under the concentration-time profile extrapolating to time of infinity, *C*_*max*_ maximum concentration, *CL* clearance, *acMMAE* monomethyl auristatin E, *NA* not available

### Immunogenicity

The prevalence of ADA at baseline was 5.3% (two out of 38 evaluable patients). Post-treatment with DFRF4539A ADAs were detected in nine of 31 patients for which post-baseline data was available. These included the two patients with baseline positive signals that were not enhanced by the treatment. Therefore the overall treatment-emergent ADA incidence was 22.6% (seven out of 31). The presence of ADA appeared to have minimal impact on the ADC exposure in this study. In patients that were ADA positive, no apparent impact on safety was observed.

### Clinical activity

**Fig. 1:**
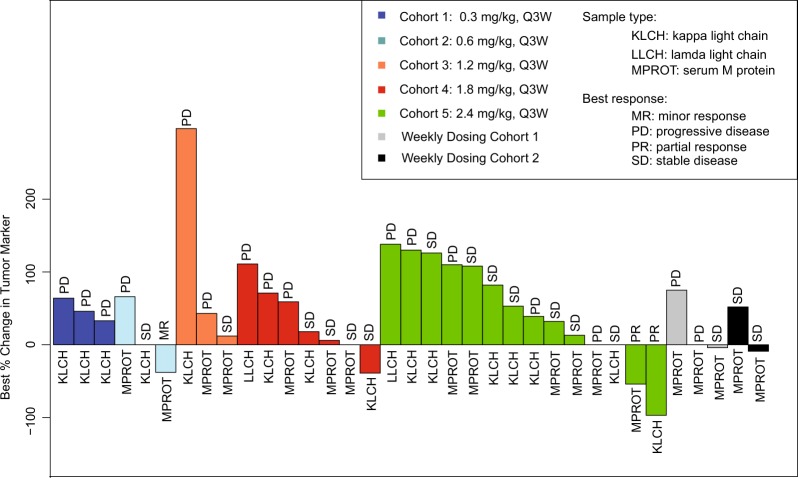
Best percent change in either serum M-protein or serum free light chain levels (in patients without detectable M-protein) relative to baseline for all efficacy-evaluable patients. Two efficacy-evaluable patients are not depicted due to lack of detectable M protein or serum free light chains at baseline; both of these patients had a best response of progressive disease

Thirty-seven patients (95%) were evaluated for tumor response (Fig. 1). Two patients (5%) had a partial response, one patient (3%) had minimal response, 18 patients (46%) had stable disease, and 16 patients (41%) had progressive disease (Table 4). The two patients with a partial response were treated at the highest dose tested, 2.4 mg/kg Q3W DFRF4539A. The duration of objective response in the two patients with PR were 22 and 66 days. Figure 1 depicts the best percent change in either serum M-protein or serum FLC levels (in patients without detectable M-protein) relative to baseline for all efficacy-evaluable patients.

**Table 4:** Investigator-assessed best overall responses

	Q3W dosing					Q1W dosing		All patients (*N* = 39)
	0.3 mg/kg (*n* = 3)	0.6 mg/kg (*n* = 3)	1.2 mg/kg (*n* = 3)	1.8 mg/kg (*n* = 7)	2.4 mg/kg (*n* = 17)	0.8 mg/kg (*n* = 3)	1.1 mg/kg (*n* = 3)	
Partial response	0	0	0	0	2 (12%)	0	0	2 (5%)
Minimal response	0	1 (33%)	0	0	0	0	0	1 (3%)
Stable disease	0	1 (33%)	1 (33%)	4 (57%)	9 (53%)	1 (33%)	2 (67%)	18 (46%)
Progressive disease	3 (100%)	1 (33%)	2 (67%)	3 (43%)	5 (29%)	2 (67%)	0	16 (41%)

### Biomarker analysis target occupancy

**Fig. 2:**
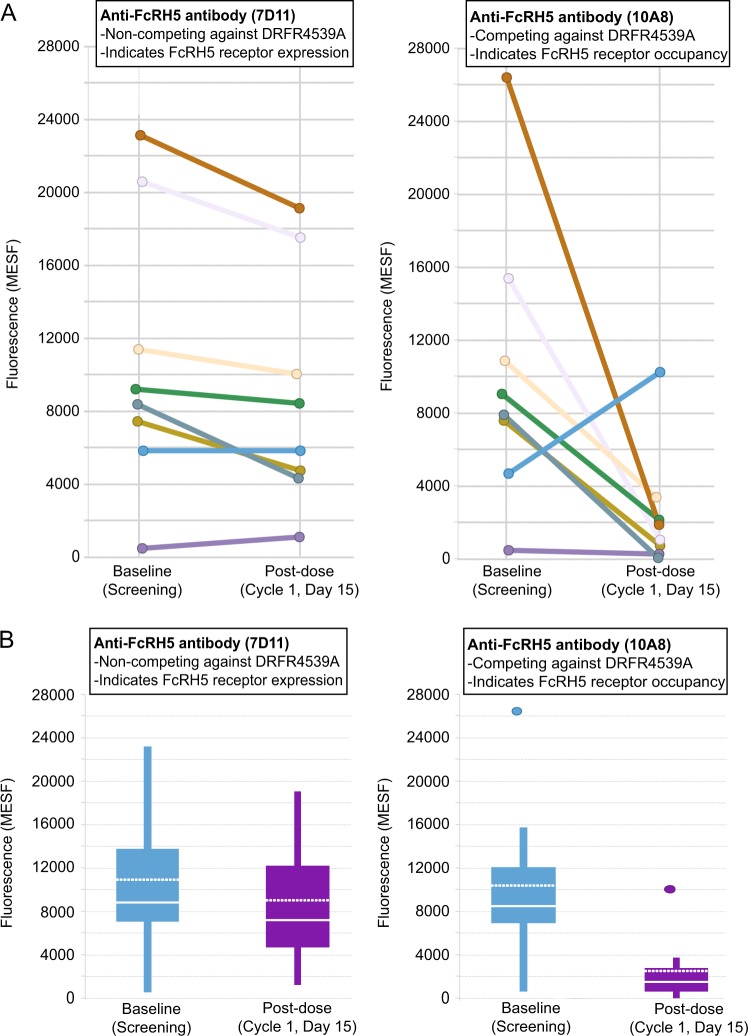
FcRH5 expression as measured by clone 10A8 and clone 7D11 in patients at baseline and post-dose. **a** Spaghetti plot of baseline and post-dosing flow cytometry data for each patient showing staining intensity—measured as molecules of equivalent soluble fluorochrome (MESF)—by an anti-FcRH5 antibody pair consisting of clone 10A8, a competing and blocking antibody with respect to DFRF4539A, and clone 7D11, a non-competing and non-blocking antibody with respect to DFRF4539A. **b** Box plot summary of FcRH5 receptor expression and receptor occupancy in patient samples at pre-dose and post-dose timepoints

Two phycoerythrin-conjugated anti-FcRH5 antibody clones (anti-CD307) were used separately to stain patient samples at baseline and after dosing. Clone 10A8, which consisted of the antibody in DFRF-4539A without the drug conjugate, was used to determine the occupancy of the FcRH5 receptor before and after dosing and was a competing and blocking antibody, whereas clone 7D11 was a non-competing and non-blocking antibody with respect to DFRF4539A for binding to FcRH5. The resulting fluorescence intensity units (MESF) for each antibody was plotted against each other for each patient sample at screening and found to be highly correlated (Pearson correlation coefficient = 0.8), demonstrating comparable binding of their respective targets (data not shown). When patient samples at baseline and post-dosing were analyzed using the non-competing antibody clone 7D11, the median MESF values were similar (baseline: 8793.0; cycle 1, day 15: 7181.5), thereby equating the level of FcRH5 in the patient population at baseline vs. post-dosing (Fig. 2). When patient samples at baseline and post-dosing were analyzed using the competing antibody clone 10A8, the median MESF value at baseline (8483.5) was comparable to that of clone 7D11 as expected. However, the median post-dosing MESF value of clone 10A8 (1515.5) was significantly lower in comparison to its baseline value, thereby quantitatively demonstrating receptor occupancy in post-dosing patient samples. Receptor occupancy was estimated to be 62.57% (*n* = 8) (Fig. 2).

**Fig. 3:**
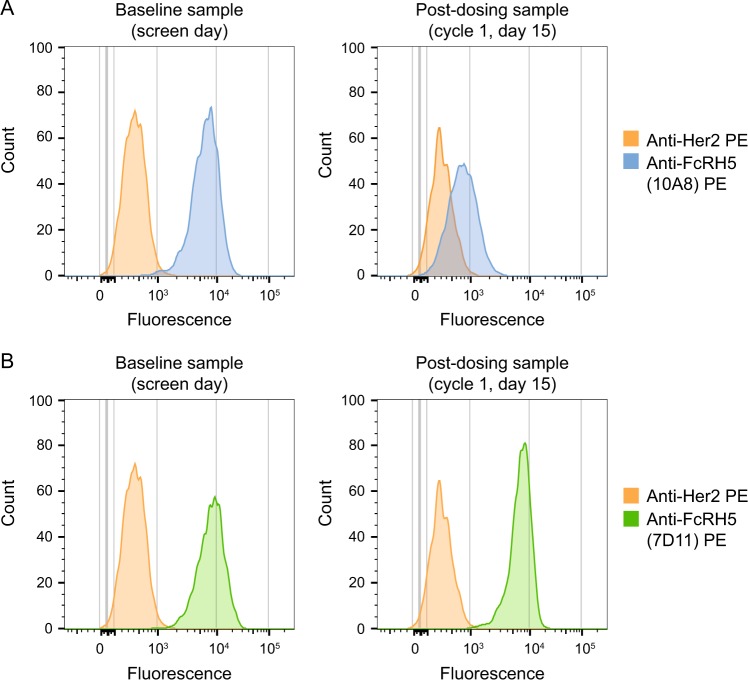
Expression of FcRH5 receptors (CD307) and DFRF4539A engagement on plasma cells (CD45^dim^/CD38^++^/CD138^+^) in bone marrow aspirates. **a** Overlay of isotype control antibody (anti-Her2) and anti-FcRH5 antibody (clone 10A8, a competing and blocking antibody with respect to DFRF4539A) at baseline vs. post-dosing. **b** Overlay of isotype control antibody (anti-Her2) and anti-FcRH5 antibody (clone 7D11, a non-competing and non-blocking antibody with respect to DFRF4539A) at baseline vs. post-dosing

Anti-FcRH5 expression levels on gated PCs in patient samples varied from 19 to 100%. Representative flow cytometry data plots that demonstrate FcRH5 receptor occupancy and expression on PCs after 1 cycle of anti-FcRH5 therapy are shown in Fig. 3. At screening, positive staining relative to isotype control was observed on PC using anti-FcRH5 antibody clone 10A8, which competes with DFRF4539A for binding to FcRH5. On cycle 1, day 15, FcRH5 receptor occupancy by DFRF4539A was demonstrated by a downshift of staining by antibody clone 10A8 relative to isotype control on PCs (Fig. 3a). In contrast, there was no observed loss of staining between screening and cycle 1, day 15, by the anti-FcRH5 antibody, clone 7D11, which is non-competing and non-blocking with respect to DFRF4539A (Fig. 3b). This demonstrated that expression of FcRH5 receptors was maintained on PCs after DFRF4539A treatment and that DFRF4539A remained bound to FcRH5 at the cell surface.

**Fig. 4:**
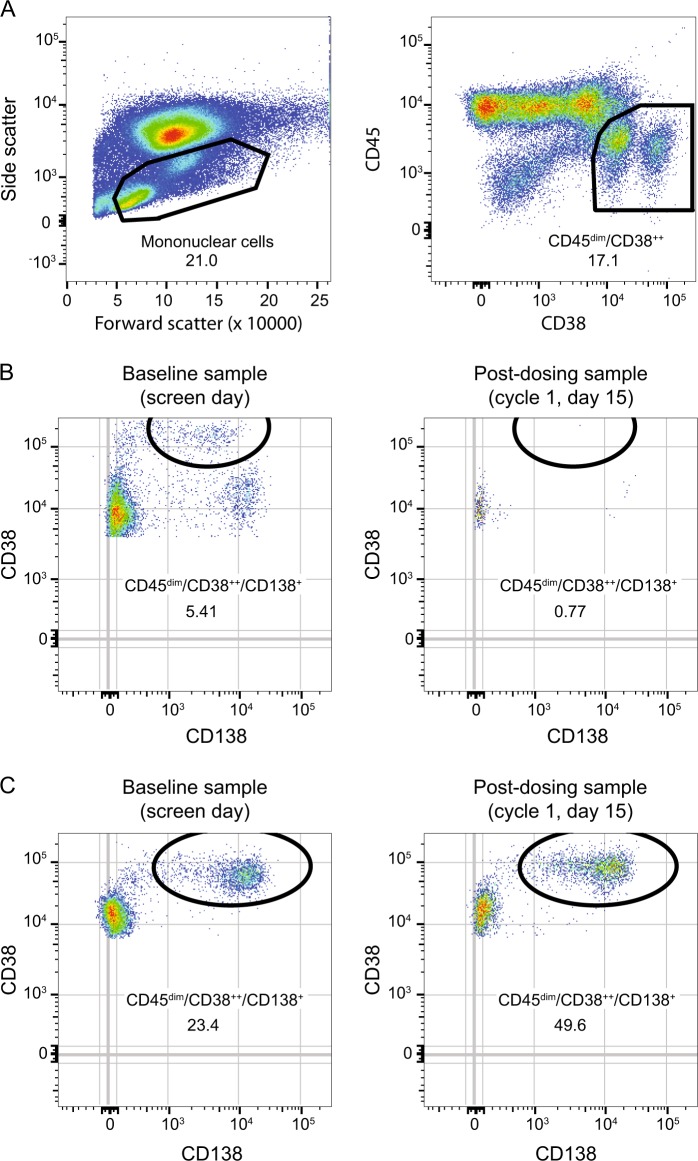
Presence of plasma cells (CD45^dim^/CD38^++^/CD138^+^) at baseline vs. post-treatment. **a** Gating strategy. **b** Representative patient with plasma cell depletion following dosing with DFRF4539A. **c** Representative patient without plasma cell depletion following dosing with DFRF4539A

PCs were not consistently depleted after anti-FcRH5 treatment (Fig. 4). In comparing baseline (screening samples) to post-dosing (cycle 1, day 15 samples), some patients showed depletion of PC (representative patient data, Fig. 4b) while other patients did not show depletion of PC (representative patient data, Fig. 4c).

## Discussion

Administration of DFRF4539A in patients with previously treated relapsed or refractory multiple myeloma was found to have acceptable tolerability, but demonstrated limited activity at the dose and schedule tested with this formulation. Target study drug exposures were achieved. There were no significant safety events considered related to DRF4539A, and there were no grade 5 AEs. The SAE reported with highest incidence overall was grade 3 acute renal failure (*n* = 2; 5.1%) at the 2.4 mg/kg Q3W dose level, and this was the only SAE found to be related to the study drug. All patients discontinued from the study largely because of disease progression, AEs, or discretion of the investigator. Overall, the rate of discontinuations due to AE’s at the 2.4 mg/kg Q3W dose level was 29%, considered high in comparison to other ADC programmes at 5–10%.

The exposure of the total antibody and the ac-MMAE analytes was linear across the tested dose range of 0.8–2.4 m/kg for both the Q3 and the Q1 schedules. Systemic exposure to unconjugated MMAE was consistently low, suggesting that majority of MMAE remained conjugated to the ADC in circulation. The T_max_ of unconjugated MMAE was delayed as compared to ac-MMAE, suggesting formation-rate limited kinetics, which was consistent with the mechanism of action for an ADC. Minimal accumulation was observed for the ac-MMAE, total antibody and unconjugated MMAE analytes upon repeated dosing for the Q3 and Q1 schedules, suggesting steady state was reached within cycle 1.

The primary clinical toxicities observed with ADC’s that contain microtubule inhibitors include bone marrow toxicity and peripheral neuropathy^23^. Bone marrow toxicity is expected due to the effect on rapidly proliferating cells in that compartment. As such, neutropenia is a commonly reported AE seen with MMAE, and neutropenia rates observed in this study are consistent with rates observed in studies of other MMAE-conjugated ADCs^24,25^. In the current study, anemia was the most common AE, and thrombocytopenia was also observed. Microtubule inhibitors also have an effect on peripheral nerves due to the long projections of axons and the critical role of the microtubule network in the nerve cell for axonal transport^26^. Peripheral neuropathy is recognized as a class-effect of microtubule inhibitors and can be one of the most frequent treatment-related AEs, ranging from 20% to 56% with the use of conventional MMAE ADCs^27^. Peripheral neuropathy (*n* = 4; 10%) and peripheral sensory neuropathy (*n* = 4; 10%) were reported in the current study.

FcRH5 during disease progression has been demonstrated by others to be consistently expressed on malignant PCs from patients with multiple myeloma^9,10^. In the current study, we used flow cytometry on pre-dose vs. post-dose patient peripheral blood samples and demonstrated target engagement of FcRH5 by DFRF4539A, and maintenance of FcRH5 expression in patient samples post-dosing. Others have demonstrated that 49% of multiple myeloma patients present with high levels of soluble FcRH5^6^. While high soluble antigen levels are a plausible barrier to therapy, we found that this did not impact PK in this patient population.

Despite achieving target drug exposures in patients, response rates in this patient population were generally low, with 5% PR, 46% SD, and 41% PD. The low activity of DFRF4539A observed in this study may be due to several factors. While the expression of FcRH5 on malignant PCs is increased in multiple myeloma patients in comparison to control PCs from normal controls^8^, the threshold required for ADC activity in the patient population is unknown. It is possible that DFRF4539A cannot kill multiple myeloma cells expressing endogenous levels of FcRH5. Additionally, levels of soluble FcRH5 are known to be elevated in the blood of multiple myeloma patients^6^, and this shed form of FcRH5 may have resulted in decreased binding of DFRF4539A to membrane-bound FcRH5. Finally, the degree of internalization of DFRF4539A by multiple myeloma cells was not directly assessed in this study. Therefore, it is possible that sufficient MMAE was delivered to the cell surface of multiple myeloma cells, but intracellular MMAE concentrations were insufficient to kill these cells.

While historical response rates to single agent MMAE is ~10%, multiple myeloma cells typically have a low proliferative index (Ki67 median 4.4%)^28^, and therefore cell-cycle dependent drugs such as MMAE conjugates may be less effective for this cell population. Microtubule inhibitors, including vinca-alkaloids, have been shown to have limited activity in multiple myeloma^29,30^. However, an ADC carrying a microtubule inhibitor payload and targeting the myeloma-associated antigen BCMA has recently shown promise in heavily-pretreated multiple myeloma patients^31^, and thus the role of microtubule inhibitors in future of myeloma therapy is uncertain.

Given the small sample size, data from this study has limited statistical power. Further investigation of DFRF4539A has been stopped due to the limited activity observed in this study. However, it should be noted that approximately 50% of patients on study appeared to derive some clinical benefit (achieving either SD or a PR). Hence, FcRH5 may be a valid myeloma target, although using an MMAE-based ADC may not be an optimal strategy to target FcRH5. Drugs with other mechanisms of action, including T-cell directing, bi-specific antibodies, or chimeric antigen receptor-modified T cells, may prove beneficial to multiple myeloma patients in the future, when directed against FcRH5.

## Supplementary information

Supplementary Information.
