# Supplementary material for: Phase I study of the anti-FcRH5 antibody-drug conjugate DFRF4539A in relapsed or refractory multiple myeloma
Source: Blood Cancer J. 2019 Feb 4;9(2):17. doi: 10.1038/s41408-019-0178-8 (PMC6362066; doi:10.1038/s41408-019-0178-8)

Phase I study of the anti-FcRH5 antibody-drug conjugate DFRF4539A in relapsed or refractory multiple myeloma

A. Keith Stewart[1], Amrita Y. Krishnan[2], Seema Singhal[3], Ralph V. Boccia[4], Manish R. Patel[5, 6], Ruben Niesvizky[7], Asher A. Chanan-Khan[8], Sikander Ailawadhi[8], Jochen Brumm[9], Kirsten E. Mundt[9], Kyu Hong[9], Jacqueline McBride[9], Quyen Shon-Nguyen[9], Yuanyuan Xiao[9], Vanitha Ramakrishnan[9], Andrew G. Polson[9], Divya Samineni[9], Douglas Leipold[9], Eric W. Humke[9], James Scott McClellan[9], Jesus G. Berdeja[6]

[1]Division of Hematology-Oncology, Mayo Clinic, Phoenix, AZ
[2]Judy and Bernard Briskin Center for Multiple Myeloma Research, City of Hope Medical Center, Duarte, CA
[3]Lurie Comprehensive Cancer Center, Northwestern University, Chicago, IL
[4]Center for Cancer and Blood Disorders, Bethesda, MD
[5]Florida Cancer Specialists, Sarasota, FL
[6]Sarah Cannon Research Institute, Nashville, TN
[7]Multiple Myeloma Center, New York Presbyterian Hospital-Cornell Medical Center, New York, NY
[8]Division of Hematology & Oncology, Mayo Clinic, Jacksonville, FL
[9]Genentech, Inc., South San Francisco, CA

Corresponding author:
A. Keith Stewart, M.B., Ch.B.
Division of Hematology-Oncology
Mayo Clinic Arizona
13400 E. Shea Blvd.
Scottsdale, AZ 85259
Stewart.Keith@mayo.edu

SUPPLEMENTARY INFORMATION

Supplementary Table S1. Adverse events related to DFRF4539A occurring in ≥ 10% of patients

	Q3W Dosing					Q1W Dosing		All Patients (*N*=39)
	0.3 mg/kg (*n*=3)	0.6 mg/kg (*n*=3)	1.2 mg/kg (*n*=3)	1.8 mg/kg (*n*=7)	2.4 mg/kg (*n*=17)	0.8 mg/kg (*n*=3)	1.1 mg/kg (*n*=3)	
Any AE	3 (100%)	1 (33%)	3 (100%)	6 (86%)	13 (77%)	2 (67%)	2 (67%)	30 (77%)
Anemia	3 (100%)	0	0	1 (14%)	4 (24%)	1 (33%)	1 (33%)	10 (26%)
Fatigue	1 (33%)	0	0	0	7 (41%)	0	0	8 (21%)
Nausea	0	1 (33%)	1 (33%)	0	4 (24%)	0	1 (33%)	7 (18%)
Increased AST	0	0	1 (33%)	1 (14%)	3 (17%)	1 (33%)	0	6 (15%)
Neutropenia	2 (67%)	0	0	0	3 (17%)	1 (33%)	0	6 (15%)
Diarrhea	0	0	1 (33%)	0	4 (24%)		0	5 (13%)
Peripheral neuropathy	0	0	0	1 (14%)	2 (12%)	1 (33%)	0	4 (10%)
Peripheral sensory neuropathy	0	0	0	2 (28%)	2 (12%)	0	0	4 (10%)
Thrombo-cytopenia	1 (33%)	0	0	1 (14%)	2 (12%)	0	0	4 (10%)

Supplementary Table S2. All serious adverse events in safety-evaluable patients.

	Q3W Dosing					Q1W Dosing		All Patients (*N*=39)
	**0.3 mg/kg (*n*=3)**	**0.6 mg/kg (*n*=3)**	**1.2 mg/kg (*n*=3)**	**1.8 mg/kg (*n*=7)**	**2.4 mg/kg (*n*=17)**	**0.8 mg/kg (*n*=3)**	**1.1 mg/kg (*n*=3)**	
Total number of patients with at least one adverse event	1 (33.3%)	2 (66.7%)	0	0	4 (23.5%)	0	1 (33.3%)	8 (20.5%)
Total number of events	2	2	0	0	4	0	1	9
Acute renal failure	0	0	0	0	2 (11.8%)	0	0	2 (5.1%)
Back pain	0	1 (33.3%)	0	0	0	0	0	1 (2.6%)
Hypercalcemia	0	0	0	0	1 (5.9%)	0	0	1 (2.6%)
Lobar pneumonia	0	0	0	0	0	0	1 (33.3%)	1 (2.6%)
Pathological fracture	0	0	0	0	1 (5.9%)	0	0	1 (2.6%)
Pneumonia	0	1 (33.3%)	0	0	0	0	0	1 (2.6%)
Pneumonia Escherichia	1 (33.3%)	0	0	0	0	0	0	1 (2.6%)

Supplementary Figure S1. Study scheme.

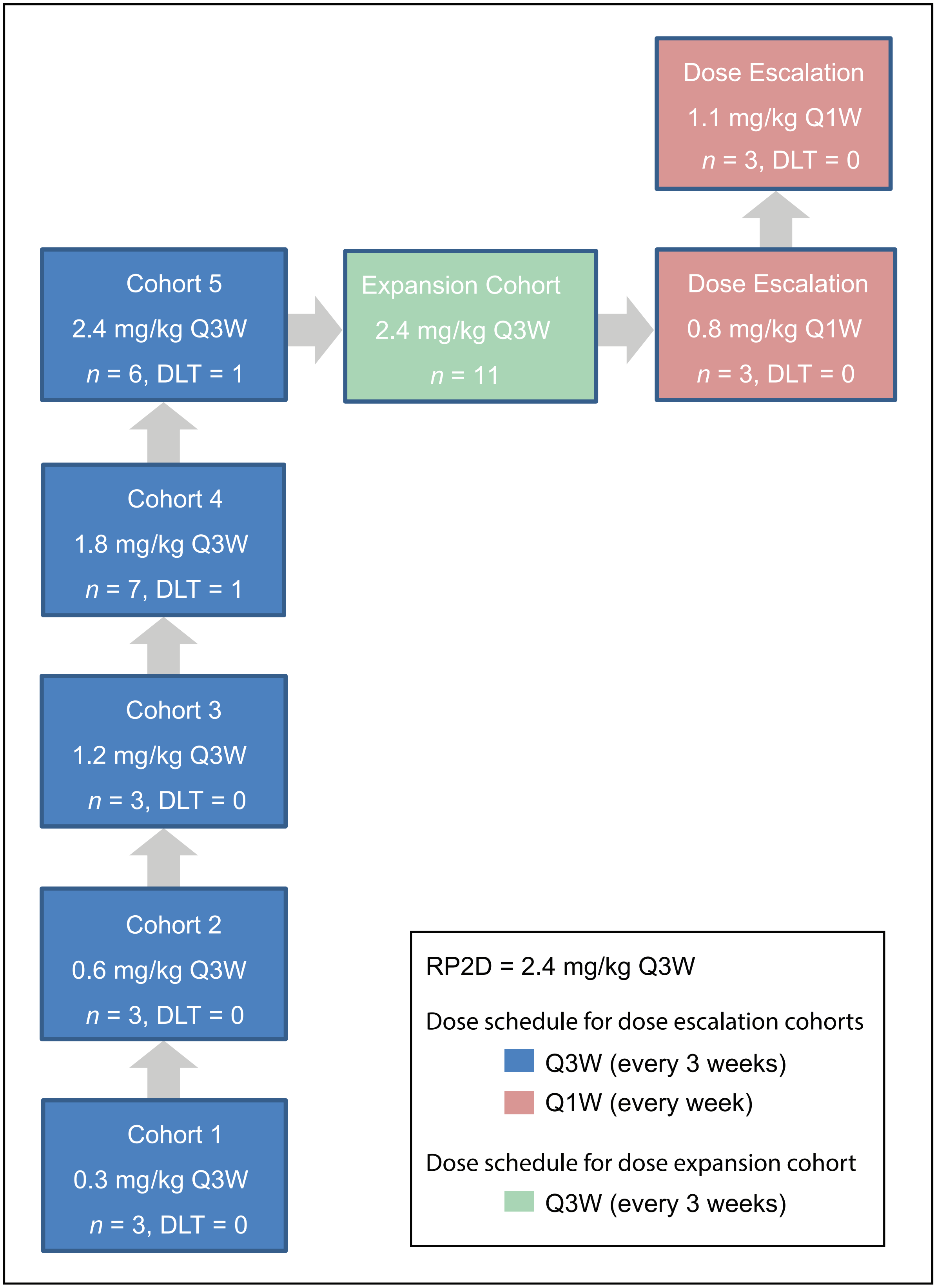

Supplementary Figure S2. Time on study for Q3W cohorts.

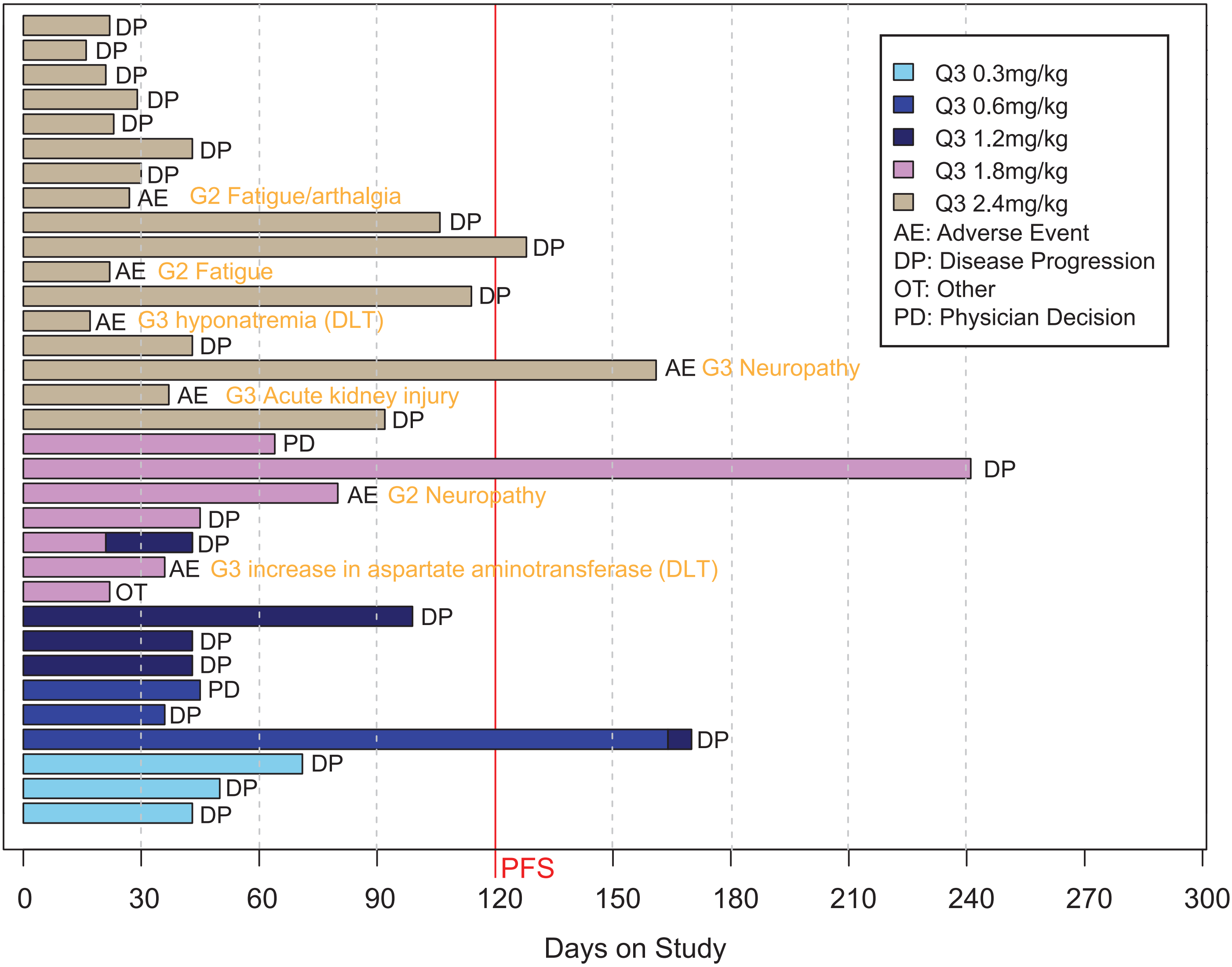

Supplementary Figure S3. Time to first onset of peripheral neuropathy in safety evaluable patients, 2.4 mg/kg, every 3 weeks dosing. Peripheral neuropathy using standardized MedDRA queries (SMQs) MedDRA v17.0.

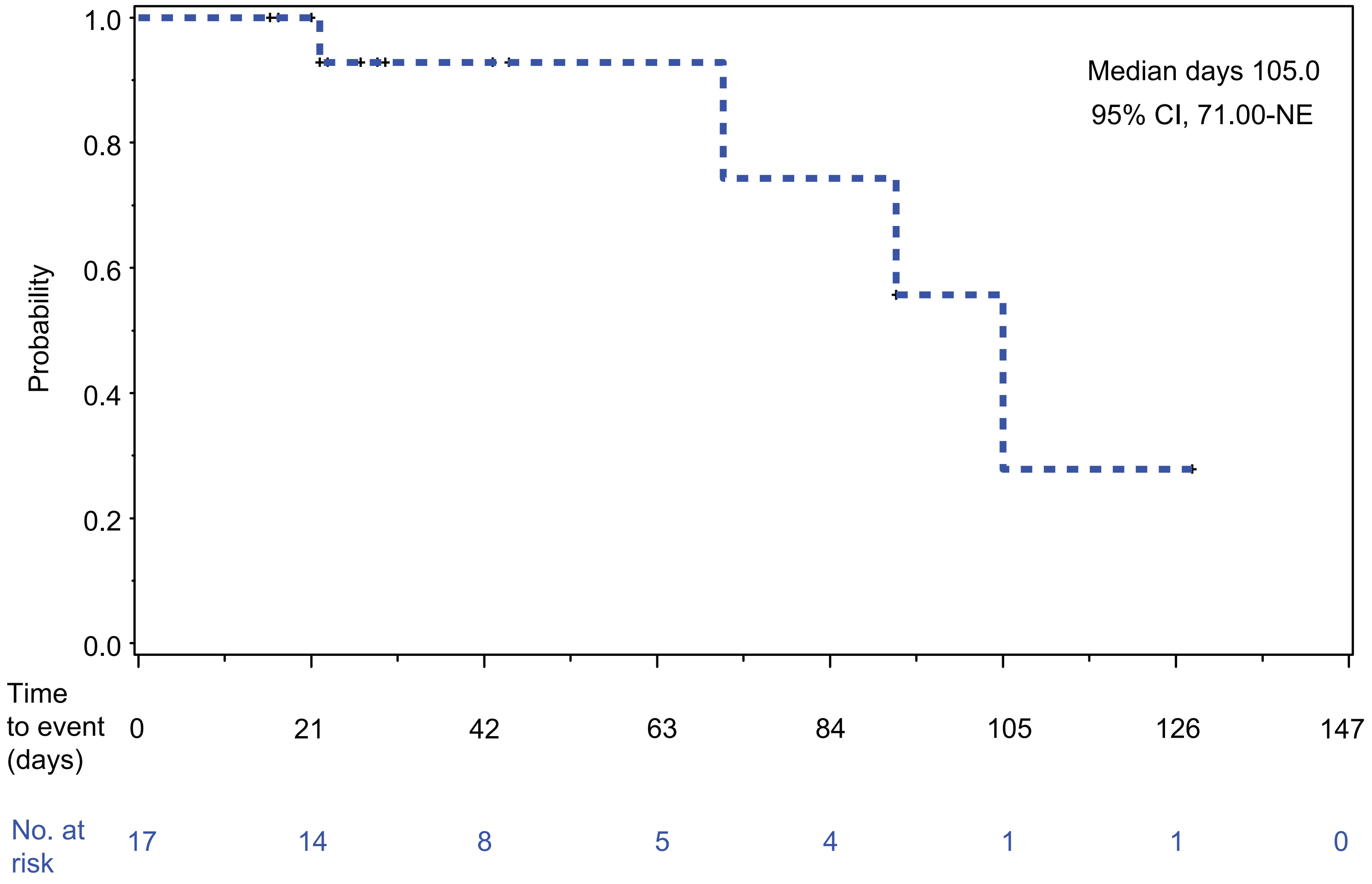

Supplement: Supplementary file 1 — Supplementary Information. [file 41408_2019_178_MOESM1_ESM.pdf]
